# Solvent-Free Ion-Conductive Xerogels with High Conductivity and Adhesion Enable Multimodal Sensing

**DOI:** 10.3390/gels11040242

**Published:** 2025-03-26

**Authors:** Yicheng Zhu, Yichen Zhou, Xing Zhang, Pengju Pan, Jinjun Yang, Chengtao Yu

**Affiliations:** 1 Tianjin Key Laboratory of Hazardous Waste Safety Disposal and Recycling Technology, School of Environmental Science and Safety Engineering, Tianjin University of Technology, 391 Binshui Xidao, Tianjin 300384, China; zyc1025759026@stud.tjut.edu.cn; 2 Quzhou Research Institute, Zhejiang University, 99 Zheda Road, Quzhou 324000, China; panpengju@zju.edu.cn; 3 State Key Laboratory of Chemical Engineering, College of Chemical and Biological Engineering, Zhejiang University, 866 Yuhangtang Road, Hangzhou 310058, China; yichenzhou@zju.edu.cn (Y.Z.); zhangstar@zju.edu.cn (X.Z.)

**Keywords:** ion-conductive gels, high conductivity, strong adhesion, flexible sensors, polyethylene glycol

## Abstract

Ion-conductive gels (ICGs) are essential for achieving human–machine interfaces, bioelectronic applications, or durable wearable sensors. However, traditional solvent-dependent ICGs face bottlenecks such as dehydration-induced failure and challenges in achieving a balance between conductivity and mechanical properties. Here, this work developed a novel ternary ion-conductive xerogel (PEM-Li ICXG) system based on polyethylene glycol (PEG), poly (2-methoxyethyl acrylate) (PMEA), and LiTFSI. PEM-Li ICXGs exhibit high conductivity (2.7 × 10^−2^ S/m), high adhesive capability (0.34 MPa), and solvent-free characteristics. Remarkably, the incorporation of ions into ICXGs simultaneously optimizes their mechanical performance. We demonstrate the application of ICGs in flexible sensors for strain or temperature sensing. The proposed synthesis strategy is straightforward and may further inspire the design of novel high-performance ICXGs.

## 1. Introduction

Against the backdrop of the rapid advancement of Internet of Things technology, the growing demand for wearable devices, miniaturized instruments, and attachable sensors has significantly driven innovative breakthroughs in flexible electronics [1,2,3]. The field has successfully developed novel solutions applicable to cutting-edge scenarios, such as biomedical monitoring [4] and bio-inspired robotics [5,6,7,8]. Notably, current electronic systems predominantly employ electrons as information carriers [9,10,11,12], which fundamentally differs from the ion-mediated conduction mechanisms in biological organisms [13,14,15]. Flexible polymer materials with ion-conductive properties through dynamic ion–polymer interactions (i.e., ion-conductive gels, ICGs) demonstrate immense potential in bridging the electromechanical performance gap [16,17,18,19]. To meet the application requirements of next-generation intelligent soft devices, such materials must integrate exceptional electrical conductivity, reliable mechanical properties, as well as environmentally responsive functionalities and other comprehensive performance metrics [20,21].

Previous research focused on constructing highly ionically conductive gels by integrating flexible polymer networks with liquid electrolytes, such as ionic hydrogels or ionic liquid gels. For instance, polyacrylamide/lithium chloride (PAAm/LiCl)-based ionic hydrogels achieve high conductivity (>0.1 S m^−1^), approaching biological tissue conductivity levels, and demonstrate potential in flexible sensing applications [22]. However, such systems heavily rely on solvation to maintain ion transport channels. Prolonged environmental exposure leads to water evaporation, causing conductive network collapse and conductivity decay exceeding 80% [23]. More critically, under dynamic cyclic strain (e.g., 100% stretching amplitude over 1000 cycles), the modulus mismatch between the liquid electrolyte and polymer network exacerbates interfacial slippage, forming millimeter-scale conductive phase-separation regions and resulting in resistance fluctuations exceeding 200% [24]. To eliminate solvent dependency, solvent-free ICXGs chemically anchor electrolyte salts within polymer networks [25]. A representative strategy involves metal-coordination systems based on polyethylene oxide and zinc bis (trifluoromethanesulfonyl)imide, where dynamic coordination between Zn^2+^ and ether oxygen groups forms crosslinked networks. This enables the material to retain an elastic modulus of 0.15 MPa at 60 °C without leakage risks [26]. However, strong coordination interactions significantly suppress Li^+^ mobility, yielding a room-temperature conductivity of only 0.017 S m^−1^, which is two orders of magnitude lower than liquid-based systems [27]. Additionally, constrained by the solubility threshold of salts in polymer matrices (typically <30 wt%), synergistic optimization of network crosslinking density and ion transport capability faces fundamental challenges [28]. Recent breakthrough studies have attempted to merge the advantages of solvent-based and solvent-free systems, such as composite electrolytes employing polyethylene glycol (PEG) and dual-network designs [29]. By dissolving lithium bis (trifluoromethanesulfonyl)imide (LiTFSI) salts in PEG solvents while forming hydrogen bonds with poly (urethane acrylate) networks, a conductivity of 0.08 S m^−1^ at 25 °C is achieved, alongside a 40% improvement in anti-swelling performance compared to conventional hydrogels [30]. Nevertheless, such systems still face mechanical limitations: when PEG content exceeds 50 wt%, the elastic modulus drops below 0.5 MPa. Although fracture elongation reaches 800%, plastic deformation readily occurs under high-frequency loading scenarios (e.g., >10 N cyclic loads in soft robotic joints), with residual strain accumulation reaching 35% after 100 cycles [31], where these limitations severely hinder the application of ICXGs [32].

**Scheme 1:**
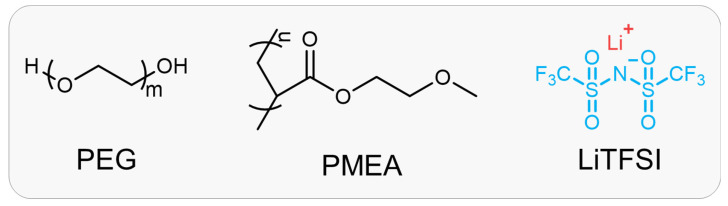
Chemical structures and compositions of PEM-Li ICXGs.

This study developed high-performance ICXGs by constructing a PEG/poly (2-methoxyethyl acrylate) (PMEA)/LiTFSI ternary system, which is abbreviated as PEM-Li. The chemical structures and compositions of PEM-Li ICXGs are presented in Scheme 1. We discovered that Li^+^ dynamic coordination establishes a reversible ion-dipole energy dissipation network [33], endowing it with a high work of extension at the fracture of 0.36 MJ/m^3^. The presence of PMEA imparts universal adhesion to wood, ceramic, and metal substrates (0.28–0.33 MPa), and LiTFSI’s dual antibacterial mechanism achieves an inhibition zone diameter of 7 ± 0.4 mm. Electrically, as an organic salt widely used in solid polymer electrolytes, LiTFSI was employed in this work to enhance conductivity. Considering the good compatibility between LiTFSI and the polymer matrix PEM, LiTFSI can dissociate as the cations Li^+^ and anions TFSI^−^ within PEM-Li ICXGs to provide charge carriers [34,35,36]. PEM-Li yields a conductivity of 2.7 × 10^−2^ S/m with dual-temperature–strain response characteristics: a temperature coefficient of resistance (TCR) of −1.54% °C^−1^ in the physiological range (30–45 °C), a gauge factor (GF) of 1.78 under 60% strain, and a response time <535 ms. After 200 cycles, the dynamic network demonstrates efficient energy dissipation and self-recovery capabilities [37]. As a multifunctional sensor, it successfully achieves synchronized monitoring of joint motion and temperature signals. We believe that this work opens a new pathway for developing high-performance ion-conductive intelligent materials [38,39].

## 2. Results and Discussion

**Figure 1:**
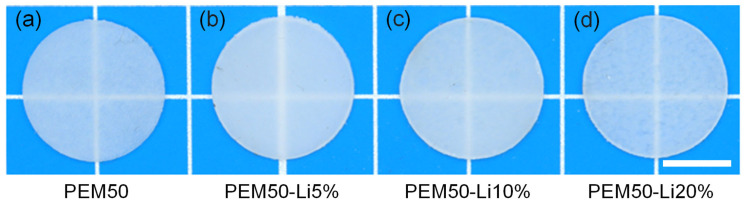
Optical images of PEM-Li membranes with varying LITFSI addition ratios: (**a**) 0%, (**b**) 5%, (**c**) 10%, and (**d**) 20%. Scale bar: 1 cm. Scale bar: 1 cm.

**Figure 2:**
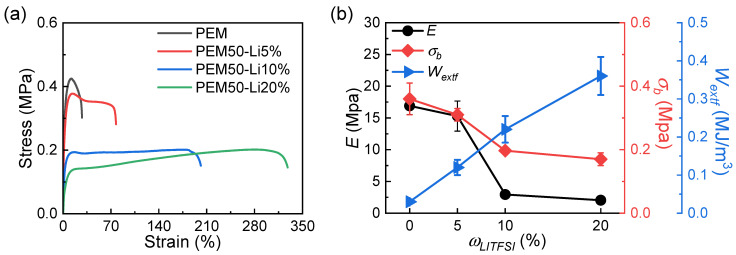
(**a**) Tensile stress–strain curves of PEM-Li ICXGs. (**b**) Mechanical properties. Elastic modulus, *E*; fracture stress, *σ*_b_; work of extension at fracture, *W*_extf_.

**Figure 3:**
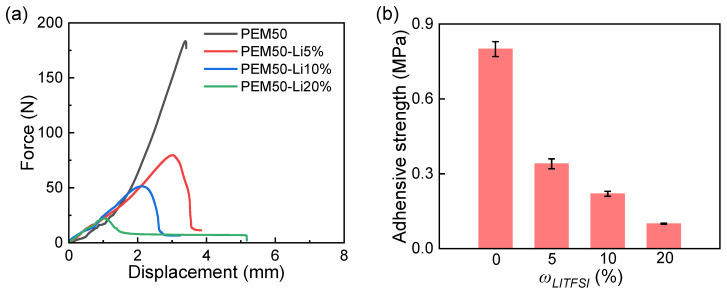
(**a**) Displacement–force curves of PEM-Li ICXGs. (**b**) Comparative analysis of interfacial bonding strength of PEM-Li ICXGs.

We successfully constructed ternary PEM*x*-Li*y* ICXGs through a free radical copolymerization strategy, where “*x*” represents the mass ratios of MEA (*ω*_MEA_) to the total MEA and PEG monomers, and “*y*” denotes the mass ratios of Li (*ω*_Li_) to the total precursor solution. The system employs PEG4000 as a flexible backbone, MEA as a functional building unit, and LiTFSI as a dynamic ionic regulation medium. As shown in Figure 1, when *ω*_Li_ increases from 0 to 20%, both the optical appearances of the PEM50 and PEM50-Li systems are turbid, suggesting distinct phase-separation phenomena formed by the PEG crystals. Of note is that the PEM50-Li samples with *ω*_Li_ ≥ 30% fail to form stable films and are excluded from further consideration. Subsequently, by systematically investigating the uniaxial tensile behavior of PEM50-Li ICXGs with *ω*_Li_ from 0 to 20% (Figure 2a), we can see that the elastic modulus (*E*) drastically decreases from 16.9 to 2.04 MPa with increasing *ω*_Li_. On the contrary, the work of extension at fracture (*W*_extf_) increases from 0.03 to 0.36 MJ/m^3^, showing a 12-fold enhancement [40]. In terms of adhesive performance, lap-shear testing results show that the PEM elastomer without LiTFSI exhibits an initial adhesive strength of 0.8 MPa (Figure 3a) due to the methoxyethyl (-OCH_2_CH_2_O-) and ester (-COO-) groups in MEA molecules forming a multi-noncovalent bonding network with the glass substrates [41]. As *ω*_Li_ increases to 5%, 10%, and 20%, the adhesive strength progressively declines in a stepwise manner from 0.34 to 0.22 MPa and finally to 0.1 MPa (Figure 3b). This degradation likely stems from two factors: (1) the competitive coordination between Li^+^ and the polar functional groups of MEA reduces the density of effective adhesive sites, and (2) the steric hindrance effect of TFSI^−^ anions weakens the physical entanglement capability of polymer chains [42,43].

**Figure 4:**
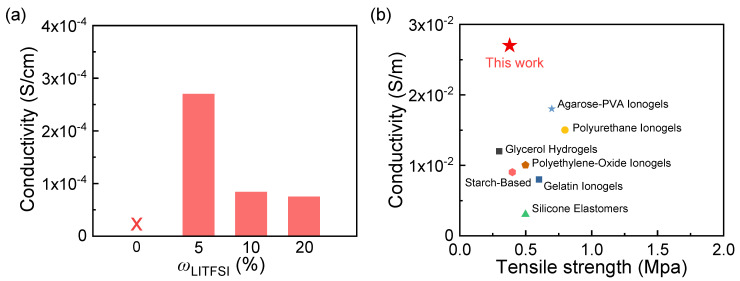
(**a**) Statistics of the conductivity of PEM-Li ICXGs. (**b**) Comparison of the tensile strength and conductivity between PEM-Li ICXGs and other reported ion-conductive materials [44,45,46,47,48,49,50].

As shown in Figure 4a, when *ω*_Li_ is 5%, the conductivity of PEM50-Li ICXG achieves the peak conductivity of 2.7 × 10^−2^ S/m, surpassing that of various ICGs reported before. It is noted that the electrical conductivity measured in this study by the DC electricity reflects the combined contributions of ionic and electronic conductivity in the material. Future work will include impedance measurements (e.g., electrochemical impedance spectroscopy) to further distinguish ionic conductivity from electrical conductivity. Specifically, its conductivity not only far exceeds that of traditional ionogels (e.g., polyurethane ionogels ~1.5 × 10^−2^ S/m, starch/gelatin-based systems ~1 × 10^−2^ S/m), glycerol hydrogels (~1.2 × 10^−2^ S/m), and silicone rubber elastomers (~3 × 10^−3^ S/m, Figure 4b) [44,45,46,47,48,49,50]. This optimization arises from the uniform dispersion of Li^+^ in the polymer matrix, forming a continuous conductive network, and the synergistic enhancement of carrier mobility through moderate ion–polymer interactions [51]. When the *ω*_Li_ exceeds 5%, it may trigger ion cluster aggregation, obstructing ion migration pathways and causing conductivity decline (Figure 4a). Based on the study of multi-parameter synergistic evolution, when the *ω*_Li_ is 5%, the PEM50-Li ICXGs achieve the relative optimal balance among mechanical, adhesive, and conductive properties.

**Figure 5:**
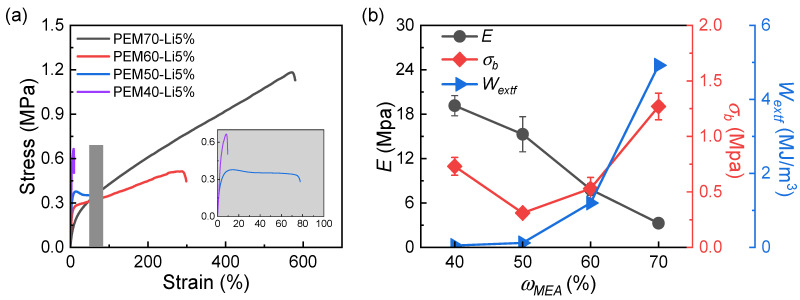
(**a**) Uniaxial tensile stress–strain curves of PEM-Li ICXGs. (**b**) Mechanical properties. Elastic modulus, *E*; fracture stress, *σ*_b_; work of extension at fracture, *W*_extf_.

Then, we examined the effects of the mass ratio of PEG and PMEA (*ω*_PMEA_) on the tensile and adhesion properties of PEM-Li5% ICXGs. As shown in Figure 5a, by investigating the influence of *ω*_PMEA_ from 40% to 70% on the uniaxial tensile behavior of ICG samples, it is found that the fracture elongation rate achieves a 40-fold leap from 15% to 580%, but the elastic modulus *E* decreases from 12.5 to 2.8 MPa, with a 77.6% reduction. This drastic mechanical transformation originates from structural reorganization involving PEG crystalline domain dissociation and PMEA dynamic network formation [52]; the former weakens the strong interchain constraints, while the latter enhances the motional freedom through chain slippage mechanisms [53]. In addition, the work of extension at fracture *W*_extf_ exhibits stepwise growth, rising from 0.05 to 4.92 MJ/m^3^, whereas the fracture stress follows a nonmonotonic trend, where it is decreasing first and then increasing as *ω*_PMEA_ >50%. The initial decline likely stems from the rigidity loss due to the PEG crystalline network dissociation, while the subsequent recovery may be dominated by phase-separation-induced crack blunting effects [54]. After comprehensive performance evaluation, the PEM50-Li5% ICXGs are ultimately selected as the optimal ratio.

**Figure 6:**
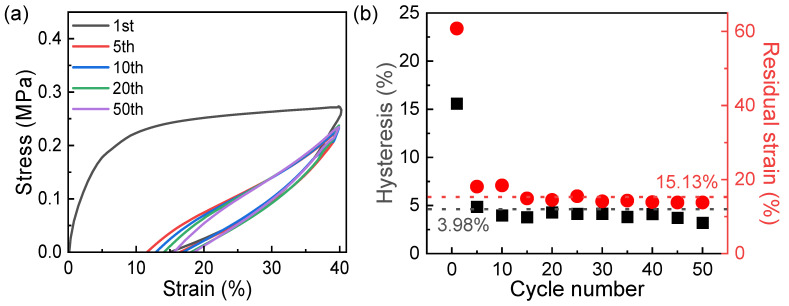
(**a**) Successive cyclic tensile stress–strain curves at 40% strain. (**b**) Hysteresis energy and residual strain.

The cyclic tensile testing results in Figure 6a demonstrate that the PEM50-Li5% ICXGs exhibit good energy dissipation characteristics during 50 cycles at 40% strain. The first cycle generates a significant hysteresis loop due to molecular chain reorganization and energy dissipation, but the hysteresis rate rapidly stabilizes around 3.98% after subsequent cycles (Figure 6b), attributed to the dynamic entanglement network achieving rapid equilibrium of energy dissipation pathways via reversible disentanglement mechanisms [55]. A residual strain of 14.33% was observed, confirming that the segmental slippage effect within the entropy–elastic network endows the materials with excellent deformation recovery [56]. Quantitative analysis of stabilized hysteresis rates and residual strain reveals the dual advantages of efficient energy dissipation and structural self-recovery in the PEM50-Li5% ICXGs.

**Figure 7:**
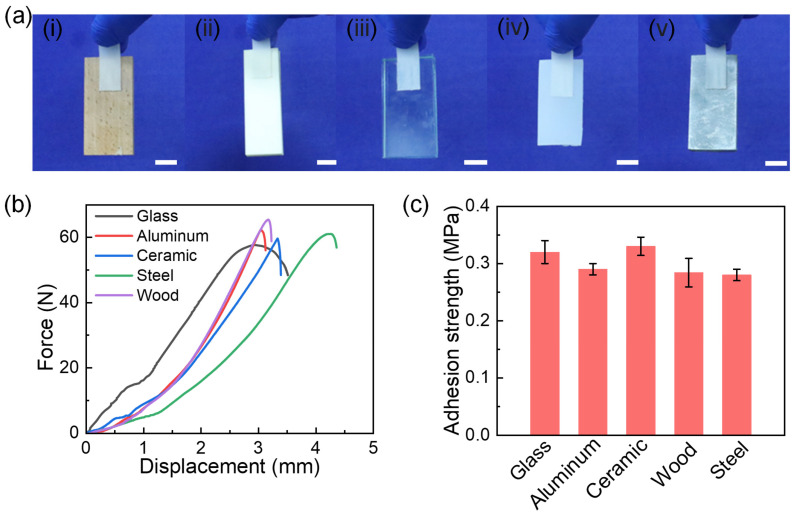
(**a**) Universal adhesion of PEM50-Li5% to various substrates (i: wood, ii: ceramic, iii: glass, iv: rubber, v: stell). (**b**) Force–displacement curves during lap-shear tests. (**c**) Adhesion strength on various substrates. Scale bar: 1 cm.

Notably, the PEM50-Li5% sample demonstrates exceptional interfacial adhesion adaptability across diverse materials. As shown in Figure 7a, experimental comparisons of its adhesive performance on substrates including wood, ceramic, rubber, glass, and metal reveal stable adhesion strengths within the range of 0.28–0.33 MPa (Figure 7b,c). This universal adhesion capability arises from the dual mechanisms of methoxyethyl and ester groups in MEA molecules: for highly polar substrates (e.g., metal, glass), ether oxygen (-O-) and ester (-COO-) groups form strong interactions with surface hydroxyls or oxides via hydrogen bonding; for low-polarity substrates (e.g., rubber, wood), the conformational adaptability of flexible polyether chains enables the accommodation of surface roughness, enhancing the interfacial bonding through chain penetration and mechanical interlocking effects [57,58,59]. The retention of such broad-spectrum adhesion, combined with the optimized electrical and mechanical properties discussed earlier, further highlights the unique advantages of PEM50-Li5% ICXGs for integration with flexible sensors and complex surfaces (e.g., bio-inspired skins).

**Figure 8:**
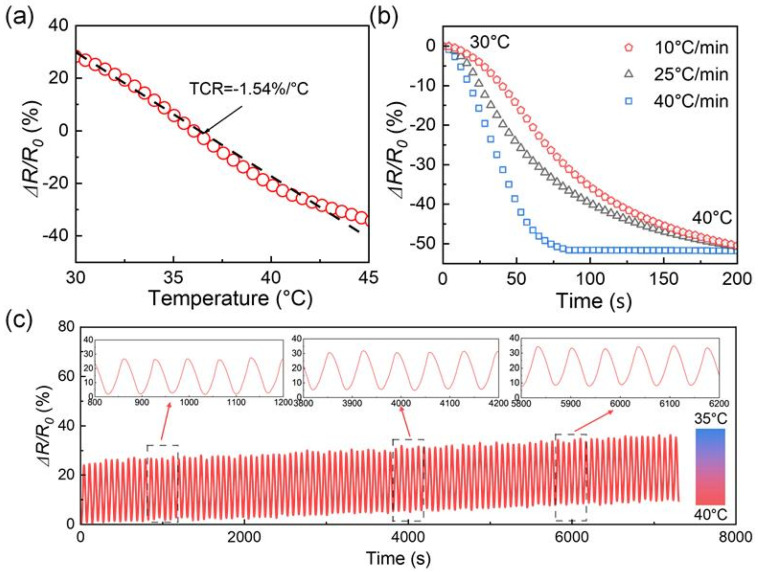
(**a**) ∆R/R_0_ changes in PEM-Li ICXGs in the temperature range of 30–50 °C. The resistance at 36 °C was taken as the reference resistance. (**b**) Comparison of temperature-resistance response under different ramp rates (i.e., 10, 25, and 50 °C min^−1^) from 30 to 40 °C. (**c**) Cyclic stability test under repeated heating–cooling in the 35−40 °C temperature range at a ramp rate of 10 °C min^−1^ for 100 cycles.

Leveraging the synergistic effects of free Li^+^ and TFSI^−^ ions in the PEM50-Li5% ICXG, it exhibits a pronounced temperature-responsive characteristic. To validate its temperature-sensing performance, we quantitatively analyzed the temperature coefficient of resistance (TCR) as a key metric [60]. Experimental data show an outstanding TCR value of −1.54% °C^−1^ at 36 °C (near human body temperature, Figure 8a), strongly confirming its potential as a temperature sensor. Further studies reveal that under varying heating/cooling rates (e.g., 10, 25, 40 °C min^−1^) within the 30–40 °C range, the final relative resistance change (ΔR/R_0_) variation remains constant (Figure 8b), demonstrating reliable electrical response characteristics. To address practical application requirements for sensor devices, we systematically evaluated the cyclic stability of PEM50-Li5% ICXGs in the 35–40 °C range. After 100 consecutive heating–cooling cycles, the ΔR/R_0_ signal amplitude remains stable (Figure 8c), with long-term stability tests fully verifying the performance reliability of PEM50-Li5% ICXGs under repeated usage conditions. The combined validation of reproducibility and long-term stability provides the critical experimental evidence for assessing the suitability of PEM50-Li5% ICXGs in biomedical temperature monitoring and related fields.

**Figure 9:**
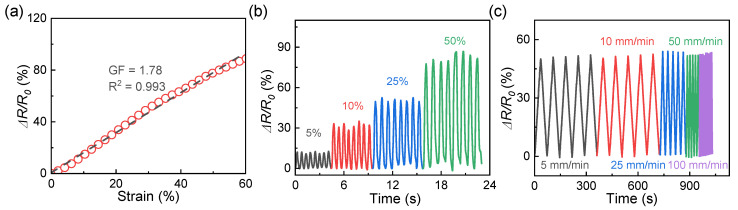
(**a**) ∆R/R_0_ changes in PEM50-Li5% ICXGs as a function of tensile strain. The red dots denote the experiment data, and the gray dashed line represents the linear fitting curve. (**b**,**c**) ∆R/R_0_ signals measured (**b**) under different tensile strains (5−50%) with the strain rate of 5 mm min^−1^ and (**c**) at different strain rates (5−100 mm min^−1^) upon stretching to 50% strain.

Likewise, systematic studies on strain-resistance response characteristics reveal that PEM50-Li5% ICXGs also exhibit exceptional strain-sensing performance (Figure 9). The PEM50-Li5% ICXGs present a high-strain responsive window from 0 to 60% (Figure 9a). The results of the gauge factor (GF = 1.78) and correlation coefficient (R^2^ = 0.993), calculated by linearly fitting the ∆R/R_0_ with loading strain, indicate the good linearity of sensing signals. Notably, this gauge factor significantly surpasses those of conventional strain-sensitive materials [61,62,63,64]. To further evaluate the practical device performance, multi-parameter cyclic testing was conducted. Under varying fixed strain levels (5–50%, Figure 9b) and loading rates (5–100 mm/min, Figure 9c), the material exhibits stable resistance responses during continuous stretch–release cycles. This high repeatability primarily stems from the three-dimensional crosslinked network structure, which endows the ICG with superior elastic recovery and microstructural stability [65].

**Figure 10:**
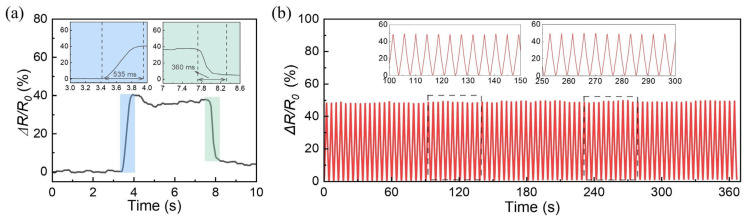
(**a**) Determination of response time and recovery time of PEM50-Li5% ICXGs at 40% strain. (**b**) Cyclic stability test under the repeated loading–unloading strain of 50% for 200 cycles. Insets exhibit the enlarged view of some periods during the whole cyclic test.

Dynamic mechanical response testing highlights the superior sensing properties of PEM50-Li5% ICXGs. As illustrated in Figure 10a, under 40% strain, the material displays exceptional dynamic responsiveness, with stretching and recovery response times of 535 and 360 ms, respectively. Notably, after 200 cyclic loading tests at 50% strain, the sensor retains stable resistance output (Figure 10b), which is a critical durability feature for dynamic monitoring applications. Combined with its sensitive temperature/strain response (Figure 9 and Figure 10), the material demonstrates significant advantages as a multifunctional wearable sensor, capable of addressing multi-parameter detection requirements in complex operational scenarios.

**Figure 11:**
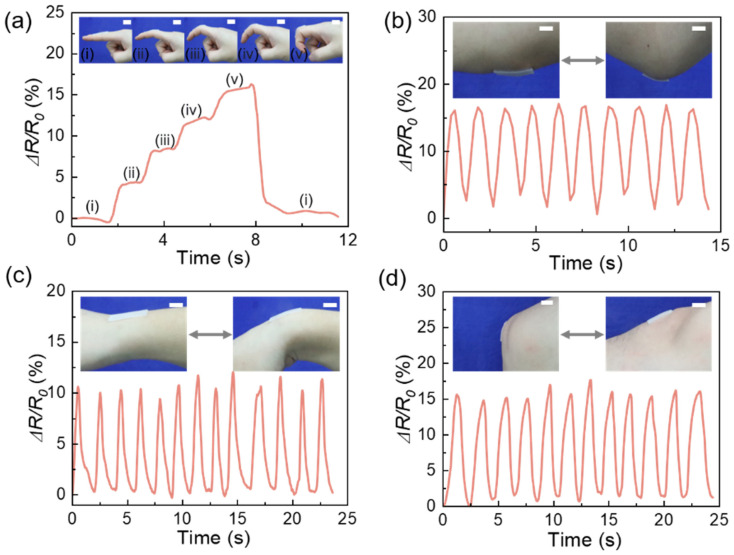
Human motion detections for the movements of (**a**) finger (i: 0°, ii: 15°, iii: 30°, iv: 45°, v: 90°), (**b**) elbow, (**c**) wrist, and (**d**) knee by the strain sensor of PEM50-Li5% ICXGs. Scale bar: 1 cm. (**a**) Human motion detections for the movements of (**a**) finger (i: 0°, ii: 15°, iii: 30°, iv: 45°, v: 90°).

The application potential of PEM50-Li5% ICXGs in biomedical sensing is particularly remarkable, as its unique temperature–strain-coupled response characteristics are fully demonstrated under human body surface temperature conditions (~36 °C). As shown in Figure 11, the PEM50-Li5% ICXGs achieve synchronous capture of multimodal biomechanical signals through flexible device integration, with core advantages manifested in two aspects: (1) stable adhesion at the material–skin interface ensures signal transmission reliability; (2) the linear mapping relationship between dynamic deformation and electrical signals enables precise motion resolution. Specifically, when deployed as a finger joint sensor (inset in Figure 11a), the device can record bending angle variations of the finger in the 15°–90° range in real time. The ΔR/R_0_ value exhibits a monotonic increasing trend with angle increments and rapidly returns to baseline within 1.2 s after reverting to the initial state (State I). Furthermore, these PEM50-Li5% ICXGs were also applied to monitor other body joints, such as the elbow, wrist, and knee joint. Figure 11b–d show that the PEM50-Li5% ICXGs can discern the flexure of the elbow joint and the bending of the wrist and knee joint.

**Figure 12:**
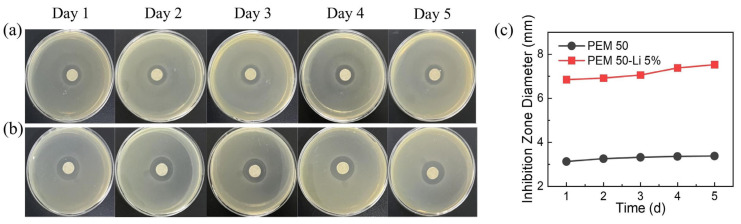
(**a**,**b**) Time-dependent variation in inhibition zones of (**a**) PEM50 and (**b**) PEM50-Li5% gels against *S. aureus* (ATCC 25923). (**c**) Comparative analysis of inhibition zone diameters between PEM50 and PEM50-Li5% gels. Scale bar: 1 cm.

In biosafety evaluation, the antibacterial efficacy of PEM50-Li5% against Staphylococcus aureus was systematically investigated via inhibition zone assays (Figure 12a). The results show that the samples with *ω*_Li_ = 5% form inhibition zones of 7 ± 0.4 mm diameter on agar plates, which is significantly larger than that of the control group without LiTFSI (3 ± 0.2 mm). After 5 days of continuous culture, the inhibition zone area of LiTFSI-containing samples remains at 108% of the initial value, while the control group maintains stable antibacterial efficacy (Figure 12b). This long-lasting antimicrobial property likely stems from the dual mechanisms of fluorinated species in LiTFSI: (1) TFSI^−^ anions penetrate bacterial cell membranes to inhibit activity; (2) gradually released Li^+^ competitively binds with phosphate groups of phospholipid head groups, disrupting membrane integrity [66]. These mechanisms collectively endow PEM50-Li5% with robust antibacterial performance.

## 3. Conclusions

In summary, this study successfully constructed PEM-Li ICXGs that leverage the dynamic coordination effects of LiTFSI to achieve synergistic regulation of mechanical, electrical, and interfacial properties. The PEM50-Li5% ICXGs have good flexibility with damage resistance while maintaining robust adhesion and high conductivity. More importantly, the PEM50-Li5% ICXGs exhibit dual-mode temperature/strain-sensing capabilities, with rapid response characteristics and cyclic stability that provide a reliable solution for human motion monitoring. The unique dynamic ionic network endows the materials with self-recovery properties and long-lasting antibacterial performance, offering a novel design strategy for the development of intelligent flexible electronic devices. Despite these promising results, challenges remain that must be addressed to facilitate real-world applications. Long-term stability under varying environmental conditions, such as humidity and temperature fluctuations, is critical for practical use. In addition, improving the environmental robustness and scalability of the fabrication process and seamless integration with existing electronic systems are essential for further advancement. Future work should focus on optimizing the material’s durability and adaptability in complex environments, while enhancing its compatibility with diverse technologies for commercialization and biomedical applications. Addressing these challenges will not only solidify the current contributions, but also pave the way for broader practical implementation and future innovations.

## 4. Materials and Methods

### 4.1. Materials

Polyethylene glycol (PEG, *M_n_* = 4000, Shanghai Macklin Biochemical Technology Co., Ltd., Shanghai, China), 2-methoxyethyl acrylate (MEA, purity 98%), lithium bis (trifluoromethanesulfonyl)imide (LiTFSI, purity 99%), dimethyl sulfoxide (DMSO, ≥99.9%), potassium persulfate (KPS), and *N*,*N*′-methylenebisacrylamide (MBAA) were purchased from Shanghai Aladdin Biochemical Technology Co., Ltd., Shanghai, China. All reagents were used without further purification.

### 4.2. Preparation of PEM-Li ICXGs

The preparation method described in this article is a universally applicable protocol, and samples with other different proportions can all be prepared through the same process. For example, polyethylene glycol (PEG, 15 g) and 2-methoxyethyl acrylate (MEA) at a mass ratio of 1:1 (PEG:MEA) were first dissolved in DMSO under a nitrogen atmosphere. The mixture was magnetically stirred at 70 °C for 1 h until complete homogenization. Subsequently, LiTFSI with 5 wt% of the total polymer mass was added in three equal portions at 15 min intervals to prevent localized crystallization. The final mixture was continuously stirred at 70 °C for an additional 1 h to obtain a transparent and homogeneous electrolyte solution. After allowing the solution to cool naturally to 40 °C, 1 wt% thermal initiator KPS and 0.2 wt% crosslinker MBAA (relative to the total monomer mass) were added sequentially. The mixture was continuously stirred for 30 min at 40 °C and 500 rpm using a constant-temperature magnetic stirrer until complete dissolution. The solution was then transferred to an ultrasonic cleaner (40 kHz) for a 15 min degassing treatment. Subsequently, the degassed solution was poured into a custom glass–rubber composite mold (100 × 100 × 1 mm^3^) and subjected to gradient polymerization in a programmable temperature oven. The polymerization protocol was set as follows: linear heating from 25 to 80 °C at a rate of 2 °C/min, followed by isothermal curing at 80 °C for 2 h to complete crosslinking. After natural cooling to room temperature, the system was demolded to obtain a white solid material with excellent ductility. Finally, the sample was placed in a polytetrafluoroethylene (PTFE) Petri dish and dried in a vacuum oven (80 °C, −0.1 MPa) for 2 h to completely remove DMSO solvent, yielding the target polymer.

### 4.3. Mechanical Property Testing

The mechanical properties of samples were evaluated using a universal material testing machine (UTM4304, Shenzhen SUNS Testing Machine Co., Ltd., Shenzhen, China) via the uniaxial tensile method. The stress–strain curve was measured to calculate the tensile strength, elongation at break, and elastic modulus of the samples. Prior to testing, the samples were cut into standard dumbbell-shaped specimens with a gauge length of 15 mm and a width of 3 mm. The ends of the specimens were clamped by fixtures, and a tensile force was applied at a constant speed of 5 mm/min until complete fracture of the specimen. To ensure data reliability, each sample was tested at least 5 times.

### 4.4. Adhesion Testing

This study employed lap-shear testing to evaluate the adhesive properties of the samples. To establish adhesive contact, the sample and substrate were first pre-compressed under 1.3 N normal pressure for 2 min to form a standardized contact interface. Subsequently, tensile loading was applied parallel to the contact surface at a constant rate of 1 mm/min until complete interfacial separation occurred. The adhesive strength was calculated by dividing the recorded maximum shear force by the pre-compressed contact area. To ensure data reliability, each experimental group underwent at least 5 independent replicate tests.

### 4.5. Electrical Conductivity Test

The bulk resistance, *R*, of PEM-Li ICXGs was detected using a two-probe measurement, with two copper electrodes attached to both ends of the samples (DMM7510 digital multimeter, Keithley, Cleveland, OH, USA). The samples were trimmed to a rectangle with a length (*l*) of 2 cm, a width (*d*) of 1 cm, and a thickness (*h*) of 1 mm and placed in a thermostatic chamber (25 °C) for 24 h before testing. The electrical conductivity σ of the samples was calculated as follows: *σ* = *l*/*Rdh*.

### 4.6. Strain-Sensing Performance Test

A strain sensor was fabricated using PEM50-Li5% ICXGs with dimensions of 25 mm in length, 10 mm in width, and 1 mm in thickness. Both ends of the PEM50-Li5% ICXGs were connected with copper wires and encapsulated with VHB adhesive as the strain sensor. The copper electrodes at both ends were then connected to a Keithley digital multimeter (DMM7510, Tektronix Technology (China) Co., Ltd., Beaverton, OR, USA) to collect real-time resistance signals. The hydrogel strain sensor was attached to human joints to monitor movement activities. The relative resistance change is defined as follows: ΔR/R_0_ = (R–R_0_)/R_0_ × 100%, where R_0_ represents the initial resistance, and R denotes the real-time resistance. The gauge factor (GF) is defined as follows: GF = (ΔR/R_0_)/*ε*, where *ε* indicates the strain applied to the sensor. It is calculated from the slope of the relative resistance–strain response curve.

### 4.7. Antibacterial Studies

Prepare a solid culture medium containing 4% glucose, 1% meat peptone broth (MPB), and 2% agar (dissolved in 100 mL deionized water). Autoclave the medium and cool it to below 40 °C. Pour 20 mL of the medium into a 90 mm sterile Petri dish and allow it to solidify for later use. Using an inoculation loop, pick a single colony of Staphylococcus aureus and transfer it to a test tube containing 10 mL of liquid MPB medium. Incubate the tube at 32 ± 2 °C in a constant-temperature incubator for 24 h to obtain logarithmic-phase bacterial culture. Mix 2 mL of the bacterial suspension uniformly into 20 mL of solid medium cooled to 40 °C. Quickly pour the mixture into a Petri dish, swirl gently to ensure even distribution, and allow it to solidify completely. Prepare PEM50-Li5% ICG discs (15 mm in diameter and 1 mm in thickness) and gently place them on the surface of the solidified agar containing the bacterial culture, ensuring full contact with the agar. Invert the plates and incubate them at 32 ± 2 °C. Observe the formation of inhibition zones every 24 h for five days. Measure the diameter (in cm) of the inhibition zones and record the data.

## Data Availability

The original contributions presented in the study are included in the article; further inquiries can be directed to the corresponding author.
